# Food Delivery Apps and Their Potential to Address Food Insecurity in Older Adults: A Review

**DOI:** 10.3390/ijerph21091197

**Published:** 2024-09-10

**Authors:** Sangchul Hwang, Cassandra M. Johnson, Joni Charles, Lesli Biediger-Friedman

**Affiliations:** 1 Ingram School of Engineering, Texas State University, San Marcos, TX 78666, USA; sanhwang@txstate.edu; 2 Nutrition and Foods Program, School of Family and Consumer Sciences, Texas State University, San Marcos, TX 78666, USA; lbfnutrition@txstate.edu; 3 Department of Finance and Economics, McCoy College of Business Administration, Texas State University, San Marcos, TX 78666, USA; jc18@txstate.edu

**Keywords:** humans, adults, aged, meals, restaurants, technology, online-to-offline services, senior nutrition

## Abstract

The proportion of older adults is increasing globally, yet many of them experience food insecurity. Technological innovations, such as increased access to internet- and mobile-based food delivery apps (FDAs), may help mitigate food insecurity. However, this topic has been understudied. This scoping review searched for publications and online technical reports from around the world using interdisciplinary databases like ScienceDirect and internet sources like government websites, respectively. Eligible references were published recently (2019–present) and focused on general technology use, including apps, among older adults (≥50 years) or FDAs for food insecurity or nutritional health generally or specifically among older adults. The search identified 19 studies from 10 countries and extracted relevant information for summary tables. A limited number of studies supported the idea that FDAs can help address food insecurity, but there are important equity considerations for older adults living in rural areas or with constrained physical abilities. Consistently, customized app features and functions increased the intention to use FDAs. In addition, FDAs may have health and environmental impacts, such as food waste and increased access or promotion of ultraprocessed foods. Additional research is needed to elucidate the potential of FDAs to address food insecurity generally and specifically among older adults.

## 1. Introduction

The proportion of older people worldwide has been increasing. Though definitions of older adults vary, the World Health Organization (WHO) has predicted that 17% of the world’s population (or 1.4 billion) will be aged ≥ 60 by 2030 [1]. In the United States (US), the older population is also growing rapidly [2]. According to US Census data, in 2020, people aged 65 and over (55.8 million) accounted for 16.8% of the overall US population, and this subgroup grew almost five times faster than the total US population between 1920 and 2020 [2]. Other countries have even higher proportions of older adults compared to the US (e.g., countries in Europe with 19% ≥ 65 years, Japan with 28.5% aged 65 years and older) [2]. Older adults are at increased risk of food insecurity and diet-related chronic diseases, because of socioeconomic factors like living alone or limited social support, having limited or low levels of income, bearing the costs of chronic disease management; and biological factors like age-related comorbidities [3,4,5,6]. However, older adults may engage with technology, including using smartphones, more than previously thought [7,8]. Technology-based solutions, such as online food delivery (OFDs) services and food delivery apps (FDAs), may be one way to address food insecurity at the population level.

The United Nations (UN) Food and Agriculture Organization (FAO) defines food insecurity as a condition that exists when people “lack regular access to enough safe and nutritious food for normal growth and development and an active and healthy life” [9]. Food insecurity is partially due to challenges with physical or economic access to food, including resources (e.g., income) [9]. The State of Food Security and Nutrition in the World 2023 indicates that 29.6% of people worldwide (2.4 billion) experienced moderate or severe food insecurity in 2022, with increases in Africa, Northern America, and Europe between 2021 and 2022 [10]. In the US, 36.7% of poor households, with incomes below the poverty level, had experienced food insecurity at some point in 2022 [11]. The US Department of Agriculture (USDA) 2023 report highlights key statistics for older adults: 9.1% of all households in the US with adults ≥ 65 years and 11.4% of households with adults ≥ 65 years and living alone experienced food insecurity at any point during the past 12 months [11]. Around the world, including in the US, food insecurity remains above pre-pandemic levels [10,11].

Numerous reviews have discussed the consequences of food insecurity for malnutrition and health, well-being, and quality of life generally and specifically among older adults [3,4,6,12,13]. The aging process introduces changes—muscle loss, reduced physical functioning, and impaired mobility—that increase the risk of noncommunicable diseases (e.g., sarcopenic obesity [3]), co-morbidities or having ≥ 1 chronic conditions, and cognitive and physical challenges that make food shopping or preparation difficult [3,12,14]. Addressing food insecurity and improving health will require technology-based solutions like online food delivery. 

**Figure 1:**
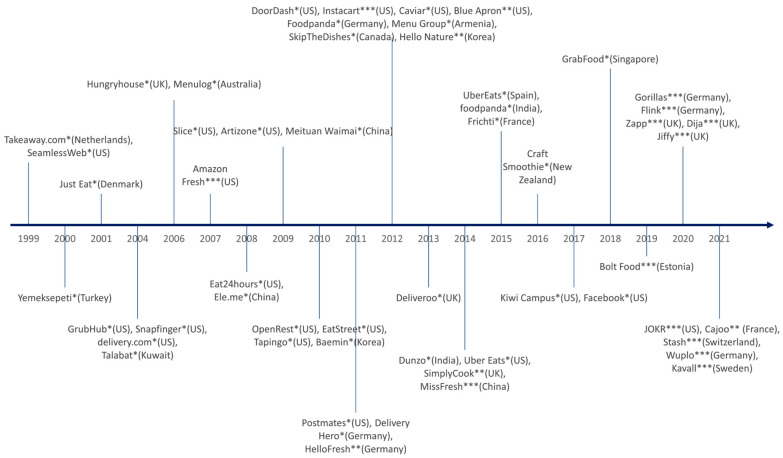
Timeline of online food delivery applications around the world by launch year. This figure shows examples of the major food delivery apps (FDAs) over time, based on public information available on company websites. The headquarter locations are shown in parentheses. Internet searches were used to identify FDAs from around the world and confirm the headquarter location. Different application types are indicated with symbols. * Meal delivery apps. ** Meal kit delivery apps. *** Grocery delivery apps.

Online food delivery (OFDs) services allow the “ordering and delivery of food from various restaurants through a website or app”, and food delivery apps (FDAs) enable users to receive foods from restaurants or third-party providers like Uber Eats [15]. Online food delivery is part of the online-to-offline services [16], which enable users to search online and order ready-to-eat foods or meals, meal kits, or groceries. The simultaneous advancement of technology—computers, smartphones, and the internet—and increase in navigation skills has increased the number of OFDs and FDAs [16]. Over the last twenty years or so, many countries in Asia, Europe, and North America have introduced OFDs and FDAs (Figure 1). Use peaked during the COVID-19 lockdowns as people who were concerned about the risk of exposure to the coronavirus could choose contactless delivery [16,17,18]. For example, online food services provided 419 million customers with more than 17 billion orders in China in 2020 [19]. The worldwide gross merchandise value of online food delivery is expected to increase to USD 1466 billion by 2027, which represents more than a 400% increase from USD 296 billion in 2021 [20]. 

In addition, emergent research indicates that older adults are interacting more with smartphones [7,8]. According to 2021 data, most older adults in the US had used the internet and owned a smartphone: 61% of adults 65 years and over had owned a smartphone; 75% of adults ≥ 65 years had used the internet [8]. The share of adults 65 years and older who are smartphone users has grown steadily between 2015 and 2021 and the differences in tech users have “narrowed” between younger and older people based on data from the Pew Research Center [8]. 

However, while there have been many recent studies published about OFDs and FDAs in general [21,22,23,24,25,26], and separately, studies of food insecurity for among older adults [6,12,13], the authors have only identified one published study specifically about technology like OFDs/FDAs as a strategy for addressing food insecurity among older adults [27]. Chiang and colleagues studied technology use and access among older adults living in the US during the COVID-19 pandemic [27], although their study did not focus on FDAs. Following the COVID-19 pandemic, food insecurity has become a critical public health issue. Older adults are at increased risk of food insecurity and related health issues [6,12], and technology-based solutions appear promising for addressing the nutritional needs of older adults [28]. The goal of this scoping review is to identify, compile, and summarize evidence from the US and around the world to assess the potential of OFDs/FDAs, referred to as FDAs going forward, as a solution for food insecurity and improved nutrition among older adults. The primary question is as follows: What are the key factors related to FDAs use among older adults? The secondary questions are the following: What is known about FDAs as an intervention strategy for addressing food insecurity generally (with adults of all ages) or specifically with older adults? What is known about FDAs as an intervention strategy for addressing nutritional health generally (with adults of all ages) or specifically with older adults? By bridging disparate bodies of literature, the findings from this review will elucidate gaps and opportunities for technology-focused interventions with FDAs among older adults globally, which is important given the increasing population and high burden of food insecurity and diet-related chronic diseases among older adults.

## 2. Methodology

This scoping review article was informed by the PRISMA Extension for Scoping Reviews guidelines [29]. A team of interdisciplinary researchers completed the review, with expertise in environmental engineering, food waste, and sustainability; public health nutrition, food insecurity, and mhealth interventions; and environmental economics. Table 1 describes the keywords, sources, and criteria used to identify relevant publications. The primary source was ScienceDirect, an interdisciplinary database for Elsevier’s publication database. ScienceDirect is well suited for this topic, which spans business, biomedical and behavioral science, and social science and compiles scholarly works from around the world. Two other publication databases, Taylor & Francis Online and Multidisciplinary Digital Publishing Institute (MDPI), were also utilized as secondary resources of peer-reviewed journal articles. Both publishers are sources of international studies. Databases were accessed through the university’s library subscriptions. Online sources were searched to identify relevant government or business reports from the United States (US) and around the world (e.g., reports by the UN). Websites for organizations, business sectors , and the US government were included in this review.

The literature search was completed using keyword combinations (Table 1). For example, one search in ScienceDirect used the following keywords: “food insecurity, food delivery application, and older adult”. The primary goal of the review was to identify FDAs for food insecurity, but a secondary and related goal was FDAs for nutritional health. The search terms were broad to capture as many relevant reports and studies as possible. In addition, the criteria for screening and eligibility were minimal to maximize the number of potential publications. For instance, the definition of older adult in this review was an individual ≥ 50 years to include more studies. There were no requirements for the research approach or study design, meaning that eligible publications have utilized quantitative, qualitative, or mixed methods approaches and varied designs. 

To determine which publications to include, the authors used two levels of review. First, one reviewer completed an initial screening using the title and abstract, which eliminated irrelevant or duplicate publications. Records that met the screening criteria were retained and assessed for eligibility using the full-text version of the document (Table 1). Second, two reviewers completed the eligibility determination. Disagreements were resolved through discussion with review team members and the authors agreed on the decision. Potentially relevant review articles were also retained for a bibliography review and used as a source of additional records. 

**Figure 2:**
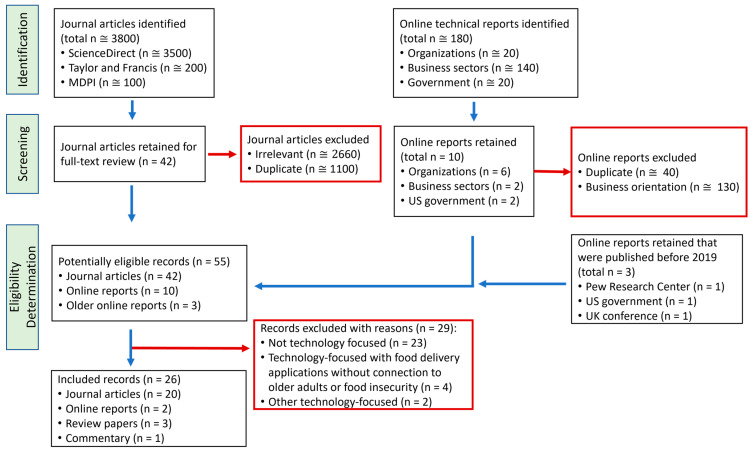
Modified PRISMA flowchart showing the identification, screening, and eligibility determination in this scoping review. The blue arrows show the process for records that moved to the next level. The red boxes and arrows show records that were eliminated. This figure was created based on a flowchart template available online [30].

A flowchart of the scoping review is shown in Figure 2 [30]. Originally, more than 3800 results were identified in the databases. For example, more than 1200 peer-reviewed original research and review journal articles were retrieved from ScienceDirect alone from 2019 onwards with tertiary combinations of keywords like food insecurity, food delivery application, and older adults. A total of 55 publications were retained from the first level of screening: 42 peer-reviewed journal articles, 10 online reports published in 2019 or after, and 3 older online reports (Figure 1). After excluding records (n = 29) that were not related to the topic and abstracts without a full-text version (n = 1), there were 26 publications, including 19 studies, related to the topic of FDAs for addressing food insecurity generally or specifically among older adults aged ≥ 50 years. For the 19 studies, key characteristics concerning the country, sample size, and type of mobile or internet-based app were extracted and organized in results tables. Two reviewers pulled information from the studies and a third reviewer checked the results tables for accuracy. The reviewers categorized countries based on economic development using the World Bank’s definitions of low-, lower middle-, upper middle-, and high-income countries [31]. 

## 3. Characteristics of Included Studies

In total, this scoping review included one commentary article [32], three review articles [26,33,34], two reports [8,35], and 19 original research studies [7,16,17,18,19,36,37,38,39,40,41,42,43,44,45,46,47,48,49]. Because the included research studies used different approaches, study designs, and methods, and as they presented the results differently, a limited number of study characteristics are presented in Table 2. All the studies utilized quantitative research approaches except the two qualitative studies that conducted in-depth interviews [36,41]. Most studies were based on cross-sectional data, with some exceptions: one study analyzed data from a 7-day period collected with ecological momentary assessment [43]; one study analyzed data from FDAs over a 305-day period [19]; two studies analyzed longitudinal data from the same individuals over time [37,47]. Bakre et al. retrospectively analyzed data from Foodsmart, a telehealth and nutrition platform with food delivery from US adults over time [37]. Wan and colleagues analyzed data from the US-based Health and Retirement Study [47], a repeated cross-sectional design that collects data every 2 years. Regarding the settings, studies were conducted in the US and around the world (n = 10 countries total): Australia, Austria, Brazil, China, India, Malaysia, Norway, Republic of Korea, Sri Lanka, and the US. Most of the included studies were from upper middle- (n = 5 studies, e.g., Brazil, China) or high-income countries (n = 10 studies, e.g., Republic of Korea, US); four studies were from lower middle-income countries (e.g., India and Sri Lanka); no studies were from low-income countries. Only three studies focused on older adults, meaning they sampled 100% older adults ≥ 50 years old [7,47,48]. The other 16 studies sampled older adults in different proportions. Details of the older adult subgroups are shown in Table 2. Most studies (n = 13) reported on FDAs; one study reported on a telehealth and nutrition platform with food delivery [37]; one study reported on a food waste app [36]; four studies did not report on a specific type of app [7,41,42,47]. Two studies, with a focus on FDAs for food insecurity, were conducted at the community-level, not the individual level [19,49]. Table 3 shows the included studies by topic.

### 3.1. Key Factors Related to Technology and Apps among Older Adults

This scoping review identified one review focused on the intention to use technology among older adults [48], two reports on general technology use for older adults [8,35], and three recent studies focused on technology use or internet- or mobile-based apps, including delivery services, among older adults [7,41,47] (Table 3). Based on the literature, the ability of older adults to use technology, including FDAs, depends on individual-level psychological factors (e.g., self-efficacy, attitude), technological factors (e.g., consumers’ perceptions about technology, intentions to use), social factors (e.g., social norms), and personal factors (e.g., age, physical and cognitive abilities) [34]. Based on their qualitative study (n = 24 adults aged 23–55 years) in Sri Lanka, Apostolidis et al. concluded that the motivations to use an app depended on personal and economic benefits and emphasized the importance of aligning the goals of various stakeholder groups to make app use more valuable to all [36]. Regarding older adults specifically, cognitive impairments or physical limitations may make it difficult to engage with technology [47]. A study in Australia explored agism-free design through in-depth interviews with technology non-adopters, aged 69–88 years and found that older adults may also have constrained access to smartphones or internet or less experience, which may limit their confidence or willingness to engage with technology and use FDAs [41]. Non-technology users felt the technology was designed for other ages of people consistently, based on age-based stereotypes [41]. A Norwegian survey assessment of older adult smart phone users found that adults 60 years of age and older had varied uses for smartphones, with social media and news being the most common; the results suggested positive self-regulation of technology usage [7].

### 3.2. Use of FDAs among Adults, Including among Older Adults

There was one study specifically about FDAs for older adults [48] out of 11 studies (Table 3). Yap and colleagues surveyed respondents ≥ 60 years in Malaysia and examined the drivers of use, adoption and access to technology among older adults by introducing them to online grocery shopping (OGS) that allowed them to shop for food without having to go to a physical store [48]. The authors found that older adults who were less able to physically function or who had lower physical mobility were more resolved to use the app. Another driver of the intent to use the app was if older individuals perceived the app as being useful and easy to use. Likewise, if users perceived that the app would improve their satisfaction with their overall quality of their life, or that the app would provide an overall improvement in the positive affective elements of their life, they would be more likely to adopt and use the app [48]. Similar motivators were found in a study by Kaur et al. that used focus groups to generate a set of questions for FDA users in India aged 25–65 years of age [40]. This study found that a consumer chooses an app for its functional and performance attributes. Further, the authors reported that behavioral values motivate a consumer’s choice to use an app to order food online and be satisfied with the delivered food. These are the social, emotional, and psychological values that would motivate a consumer’s choice and use of an app [40]. Based on their qualitative study (n = 24 adults aged 23–55 years) in Sri Lanka, Apostolidis and colleagues concluded that the motivations to use an app depended on personal and economic benefits and emphasized the importance of aligning the goals of various stakeholder groups to make app use more valuable to all [36]. Using data from Brazilian adults, collected from adults 18 to ≥ 60 years, during the COVID-19 pandemic, Zanetta and colleagues found that 37% used FDAs at least once a week, and most (76%) used FDAs for dinners on weekends [18]. Their findings underscored the importance of convenience, habit, as well as solidarity, as significant factors in the continuance intention of technology use. They also noted important differences across regions within the country [18].

Tandon et al. surveyed adults in the US (21–60 years) and found several precursors to an FDA user’s value-based behavior [16]. The prominence, or heightened awareness of, the app, through advertising, promotion, and peer use, and the user’s attitude underlie consumption values, such as food quality and functional values related to the intent to use the app and make a purchase [16]; the conclusions stressed the role of attitude in the intent to purchase with FDAs [16]. In a study of restaurant delivery services in the US before and after the COVID-19 pandemic, respondents to the stated-preference survey only had to be 18 years of age and older [39]. Respondents named safety, affordability, convenience, efficient (on-time) delivery, accurate delivery and temperature-controlled food delivery as factors at the top of their wish list [39]. Kaplan et al. found issues with equity in relation to food accessibility via FDAs, such as people in more populated areas feeling more satisfaction with food delivery compared to those living in less populated areas [39]. Additionally, adults 45–54 years and ≥ 55 years were less likely to use FDAs versus younger aged adults 18–44 years before, during, and after the pandemic [39]. In Australia, researchers administered a cross-sectional survey to adults (≥ 18 years) and determined whether meals delivered using online meal-ordering services were a healthier alternative than home-cooked meals [38]. They found that younger people with a greater body mass index and higher education levels were more likely to have engaged in ordering online. Liu et al. conducted a study in China and asked adults about what, how often, and how much they purchased when ordering online [42]. Food quality—safety, freshness—as well as price, delivery reliability and speed, the food delivery app’s ease of use, reliability, and technical support rounded out the customer’s satisfaction with the app [42]. App features, such as the food presentation (e.g., plate size), play an important role in how much food is ordered and subsequently eaten or wasted. Trivedi et al. showed the importance of app features that enable users to estimate food quantities and minimize over-ordering and food waste [46]. These attributes are likely important to older adults, too. 

Overall, there are some common themes in the research studies examining FDA use and benefits, even if the target of these studies was not only older adults. Studies showed that the features of the app (e.g., ease of use, app reliability), food (e.g., relative affordability, quality or freshness), and delivery experiences (e.g., reliability, timely) were key factors. Findings from the studies highlighted key opportunities for equity in engaging older adults with FDAs, considering educational attainment, access to technology, and digital literacy [38,39,48].

### 3.3. The Potential for FDAs to Address Food Insecurity or Nutritional Health

As shown in Table 3, four studies focused on food insecurity, including sustainability and resiliency [19,36,37,49], and one study focused on FDAs and nutrition-related behaviors like disordered eating urges [43]. Unfortunately, none of the studies of food insecurity or nutritional health focused exclusively on samples of older adults. Regarding food insecurity, only one of the included studies reported on a nutrition and telehealth platform with food delivery feature [37]. Other studies used surveys or geospatial techniques to collect individual- or community-level data related to apps for food insecurity [19,49]. 

Using data from adults across the US, with about 15% aged ≥ 60 years, Bakre et al. documented significant opportunities to alleviate food insecurity and enhance diet quality and health outcomes through an online telehealth and nutrition platform [37]. The Foodsmart app has six features: CookItNow, recipes, meal planner, food buying (transfers a user’s customized meal plan to a food retailer for online grocery pickup or delivery), deals, and telenutrition [37]. Their study was the first longitudinal study evaluating intervention outcomes, although they were not able to determine how Foodsmart app usage correlated to outcomes like food insecurity [37]. 

Wang and colleagues reported a positive contribution of FDAs to food resiliency in urban China during the COVID-19 pandemic [19]. Their study analyzed data from 57 cities over 305 days during the lockdown and reopening periods of the pandemic. The results showed that the online food delivery platforms enabled restaurants to provide needed foods to residents [19], which supported food security. Specifically, Wang et al. reported increases in the average transaction values (total value and net value after delivery fees) and orders per restaurant, as well as increases in the number of orders for online food delivery after lockdown; these were indicators that online food delivery continued to grow one year later [19]. Changes were seen in the kinds of food ordered with online food delivery during and after reopening: Wang et al. also reported a decrease in orders of Chinese and Western foods, with more processed foods, and an increase in orders of fresh food during lockdown period. Overall, their study demonstrated a unique role of FDAs in a resilient food system [19]. 

In another study completed in urban China, Zhang et al. generated novel findings about the role of FDAs in the equity of food accessibility [49], which bolsters FDAs as a potential tool for addressing food insecurity. They analyzed geo-spatial data from one city with urban, suburban, and rural districts (n = 1219 communities), including socioeconomic indicators related to equity (e.g., age and income). At the community level, between 3.8% and 50.4% were seniors aged ≥ 60 years [49]. The findings showed a positive relationship between online food delivery and food accessibility regardless of the transport mode, and that suburban and rural communities had greater access to restaurants with online food delivery [49]. The authors noted that rural communities usually had the lowest food accessibility scores regardless of the type of transportation, so while food accessibility improved, the disparity between urban and rural communities increased [49]. Importantly, their study concluded that communities with larger proportions of older adults were more likely to face low food accessibility and suggested that older adults may not have the resources or opportunities to utilize online food delivery (e.g., internet use as part of lifestyle, digital literacy) [49]. 

The positive contributions of FDAs to food security seen in studies in China need to be considered in context. China has widely available and affordable internet technology, relatively low costs of labor, highly concentrated urban populations located close to restaurants with access to transportation, and high costs of at-home food preparation that may have facilitated the success of FDAs [19,49]. 

A qualitative study about food waste apps for food security found that both consumers and food businesses valued an app that facilitated easy access to affordable food, but discrepancies in goals across stakeholders might make sustainable value co-creation difficult [36].

Regarding nutritional health, no included study of FDAs was focused on older adults. However, one study reported on FDAs and eating behaviors for a general population of adults in Australia and their sample recruited older adults [43]. Using ecological momentary assessment to collect data from FDA users and non-users over a 7-day period in Australia (adults 18–76 years old), Portingale and colleagues reported FDA use as a statistically significant predictor of urges for overeating compared to non-use of FDAs for the entire sample (n = 483) [43]. This study sampled mostly younger adults (mean age: 20.5 years) and obtained measures relevant to eating disorders [43] rather than understanding eating behaviors in context of food insecurity. 

Based on a limited number of studies, FDAs may improve access to foods for the general population [19,36,37,49], which would reduce food insecurity and potentially improve nutritional health, but this review identified no studies focused on FDAs and food insecurity specifically for older adults. Additionally, the studies included raised important concerns about the use of FDAs, such as people over-ordering and experiencing disordered eating urges [43,45], which may negatively affect nutrition.

## 4. Discussion

Recent literature provides general support for the potential value of engaging older adults with FDAs [34] or considering FDAs as a strategy for food insecurity or nutritional health [26,28,32,33]. However, leveraging FDAs for food insecurity is contingent on factors like widely available and low-cost internet and technological devices, population and food service distributions and access to transportation within a region, and the relative affordability of foods prepared at restaurants, or away-from-home foods, compared to home-prepared foods [19,49]. This review is the first, based on the authors’ knowledge, to summarize the relevant literature related to FDAs for food insecurity among older adults and offers an important contribution to the literature. 

A major finding is the dearth of studies on this topic. As others have stated, studies of FDAs have been performed from a business perspective rather than a nutrition or health perspective [26]. This review identified one study related to FDAs among older adults [48] and no studies related to FDAs for food insecurity with older adults, although there were three studies of FDAs for food insecurity among a general population of adults [19,37,49]. Only two longitudinal studies had assessed technology or FDA use among the same individuals over time [37,47], and no longitudinal studies with 100% older adults were identified on this topic. Previous review papers have emphasized the lack of longitudinal studies on food insecurity among older adults [6,12]. In addition, no studies from low-income countries were found in this review, which is like another review focusing on low- and middle-income countries but that only identified one study [6]. Studies on FDAs have mostly focused on delivery from restaurants rather than other food service providers distributing free food as part of nutrition or food assistance programs. 

Given these gaps, there is a massive opportunity to conduct research studies from a public health or nutrition perspective about technology use and FDAs among samples of older adults in the US and worldwide, particularly utilizing longitudinal designs or in countries with larger proportions of older adults or low- and middle-income countries, which were not as represented in the included studies. Additional opportunities would be research that uses FDAs to connect residents, including older adults, to the full range of food service providers, restaurants as well as community-based organizations that offer home-delivered meals to older adults. Another major finding was that FDAs can have positive impacts on food accessibility and may be potentially valuable for addressing food insecurity. One China-based study found that FDAs can have a food system stabilizing effect in urban areas [19]. The summarized studies generated important insights about issues concerning the access, use, and benefits of FDAs relevant for older adults, even though few studies explicitly focused on older adults. Kaplan and colleagues’ findings from the peri-pandemic period showed that FDAs were useful for people who had physical limitations and lived in rural areas with less access to internet or transportation options [39]. Even though older adults were not targeted in the Kaplan study [39], older adults would greatly benefit from FDAs as a solution to transportation barriers and access to safe, healthy, temperature-controlled meals and groceries. Additionally, customer loyalty or habit to use an FDA increases across all ages of adult FDA users when the app is personalized and useful, and customers are satisfied [42].

This scoping review has practical implications, such as informing future community research or outreach projects focused on access to food, including initiatives for public health nutrition or food systems. The benefits of FDAs can extend beyond nutrition, such as using FDAs to improve socialization through online (in-app) or hybrid interactions. Existing senior nutrition programs in the US, like those funded through the Older Americans Act Title III-C grants, provide home-delivered meals to eligible older adults ≥ 60 years, their spouses, and others with goals being to “(1) reduce hunger and food insecurity, (2) promote socialization, (3) promote health and well-being, and (4) delay adverse health conditions” [50]. This study’s findings may motivate the use of FDAs as part of home-delivered meal programs for older adults. The offline in-person interaction between the meal delivery person and the meal recipient is beneficial for older adults. Thomas et al. conducted a randomized study in Florida and Texas to compare the outcomes of two methods of delivering meals to older adults: home-delivered ready-to-eat meals by Meals on Wheels (MOW) and mail-delivered frozen meals, both of which provided nutritious food [51]. All the participants were home-bound with self-reported dementia. The outcome of interest was the incidence of nursing home placement. Although there were limitations to the study, the results suggested that the participants who benefited from the daily social contact with delivery persons had a lower incidence of nursing home placement. In other words, in-person home delivery of meals contributed to their social, psychological, emotional and physical needs while providing older individuals with opportunities for independence, self-efficacy and aging in place [51]. Large-scale programmatic evaluations have demonstrated the efficacy of home-delivered meal programs to improve outcomes among participating older adults [52]. Policy implications include advocating for existing nutrition programs for older adults to consider integrating technology-based solutions with FDAs and strengthening new or existing programs that provide affordable high-speed internet and electronic devices to older adults, especially in low-income communities and residents living in rural or remote areas [19,49].

A few points warrant further discussion. While this review generated insights about the use and benefits of FDAs, there are potential drawbacks of FDAs for human and environmental health [26,32]. Others have outlined the nutritional health consequences of FDAs [32], such as the relatively high amounts of total energy, saturated fat, sodium, and sugar found in restaurant foods, also described as “food away from home” [52,53]. This kind of dietary pattern, defined by the intake of ultraprocessed foods and ingredients, has been associated with negative health outcomes, like cardiovascular disease, diabetes, and cancer [54]. Prior research has shown that FDAs offer proportionately more “discretionary” food items, which are high in saturated fat, sugar, sodium and low in fiber, for purchase, and promote value bundles with discretionary foods in FDAs [55]. With FDA use, people are also exposed to advertising linked to ultraprocessed foods [56] and discretionary foods [55], and challenges estimating the sizes of menu items for online orders [46], which can constrain their autonomy and negatively impact nutritional health through purchasing behaviors. 

FDAs also can negatively impact environmental health. Previous research has described connections between FDAs and environmental concerns [33,57,58]. Environmental issues with FDAs relate to disposable food packaging, over-ordering, and wasted food [46,58] and the resources required for delivery [59]. For example, during the COVID-19 pandemic, consumers tended to order more food through the use of FDAs than needed [45]. Over-ordering through FDAs could increase plastic packages, food waste generation, and carbon emissions [33,49,57], offsetting the positive impacts on food insecurity. 

Emergent dialogue related to leveraging FDAs as a solution for food insecurity must consider equity [4,10,49]. This scoping review included more studies from upper middle- and high-income countries compared to lower middle- or low-income countries, and no studies from low-income countries. Another scoping review also found that low- and middle-income countries have not been represented in studies of food insecurity among older adults [6]. Within and across countries, older adults may have differential access to the resources and opportunities to access technology, including FDAs, including income, internet, and electronic devices. Efforts with FDAs must seek to balance social benefits with environmental costs [49]. Additional research is also warranted to understand how to equitably address food insecurity with FDAs, including differences between rural, suburban, and urban communities [49].

Ageism must also be addressed in technology-related research, including FDAs [41]. Generally, older adults have not been as involved in this kind of research compared to younger adults, given the limited number of studies that included or focused on older adults in this review. Scholarly discourse related to older adults and technology has emphasized limitations rather than existing resources and opportunities. This review calls attention to inclusion, specifically creating opportunities for adults across the life course to share in the burdens and benefits of research. Shifting from a deficit- to an asset-based framework might enable researchers and communities to work together to develop effective technology-based solutions for food insecurity and nutritional health. 

This scoping review has limitations. As a scoping review and not a systematic literature review, the authors did not conduct an exhaustive search of peer-reviewed or gray literature using multiple databases to identify research reports outside of peer-reviewed journal articles. Only one author completed the initial review. There was no assessment of the study methodology as a critique or evaluation. Due to the heterogeneity of the included studies, the data extraction in tabular form (e.g., Table 2 and Table 3) focused on a limited number of study characteristics. However, given that the topic—FDAs among older adults and FDAs to address food insecurity—has been understudied, this scoping review offers valuable insights. The strengths of this review include integrating a global focus in the literature search, with the sources of the studies being 10 countries, identified using ScienceDirect and an international publishing company, Multidisciplinary Digital Publishing Institute (MDPI), and international governmental entities, including their online technical reports. The additional strengths were the interdisciplinary team of authors conducting this review and working collaboratively to determine which publications were eligible, conduct data extraction, and interpret findings.

## 5. Conclusions

The population of older adults is growing and there are more opportunities to engage older adults with technology like FDAs to mitigate food insecurity and promote nutritional health. This scoping review bridged two bodies of literature related to technology use among older adults, with FDAs for food insecurity or nutritional health generally or specifically for older adults. Given the very limited number of studies at the intersection of FDAs for food insecurity, especially with older adults, there are many unknowns about how to leverage FDAs, as a form of technology, to address food insecurity and support nutritional health for this priority population. The future directions for research include studies examining perceptions, behavioral intentions, and behaviors related to FDAs, or the effects of FDAs on food insecurity or other indicators of nutritional health for older adults. Additional efforts are warranted to leverage FDAs to equitably address food insecurity for communities in the US and around the world for this priority population, while also considering the unintended consequences of FDAs on human health and the environment.

## Figures and Tables

**Table 1:** Keywords, sources, and criteria used to retrieve relevant publications.

Search Component	Details
Keywords	Older adult (aged ≥ 50 years), elder, food delivery apps, online delivery platforms, food insecurity, food security, nutrition, health
Databases and other resources	Publication databases: ScienceDirect, Taylor & Francis, Multidisciplinary Publishing Institute (MDPI) Internet sources: Governmental organizations (e.g., United Nations Food and Agriculture Organization (FAO), United States Department of Agriculture (USDA)), business sectors (e.g., Statistica)
Screening criteria	Published in the past 5 years (from 2019 to present) Peer-reviewed original research and review articles Full-text availability Written in English Focused on technology, including internet- or mobile-based apps, among older adults or technology or internet/mobile apps for food insecurity, nutrition, or health
Eligibility criteria	Reviews, reports, or research articles related to the topic of technology, internet- or mobile-based apps (e.g., FDAs) with older adults, or technology, internet- or mobile-based apps (e.g., FDAs) for food insecurity, nutrition, or health

**Table 2:** Selected characteristics of the included studies with older adults (≥ 50 years) about FDAs and food insecurity.

	Abbreviated Citation	Country	Sample Size (n) of Adults	Type of App
1	Apostolidis et al. 2021 [36]	Sri Lanka **	Total sample n = 24 ^a^; Older adult sample n = 4 (16.7%) aged ≥ 50 years.	Food waste
2	Bakre et al. 2022 [37]	US ****	Total sample n = 4595; Older adult sample n = 2417 (52.6%) aged 40–59 years; n = 679 (14.8%) aged ≥ 60 years.	Telehealth and nutrition with food delivery
3	Belarmino et al. 2021 [17]	US ****	Total sample n = 629; Older adult sample n = 54 (8.6%) aged ≥ 55 years.	Meal/restaurant delivery
4	Busch et al. 2021 [7]	Norway ****	Total sample n = 154; Older adult sample 100% aged ≥ 60 years, range: 60–89 years. ^b^	Not specific ^c^
5	Dana et al. 2021 [38]	Australia ****	Total sample n = 556; Older adult sample NR (46–60 years)	Meal/restaurant delivery
6	Kaplan et al. 2023 [39]	US ****	Total sample n = 423; Older adult sample n = 79 (18.7%) aged 45–54 years; n = 82 (19.4%) aged ≥ 55 years.	Food delivery (grocery and meal/restaurant delivery)
7	Kaur et al. 2021 [40]	India **	Total sample n = 423; Older adult sample NR (18–63 years)	Food delivery
8	Köttl et al. 2021 [41]	Austria ****	Total sample n = 15 ^a^; Older adult sample 100% aged ≥ 65 years, range: 69–88 years ^b^	Not specific ^c^
9	Liu et al. 2023 [42]	China ***	Total sample n = 515; Older adult sample n = 109 (21.2%) aged 46–55 years; n = 46 (8.9%) aged ≥ 56 years.	Not specific ^c^
10	Portingale et al. 2023 [43]	Australia ****	Total sample n = 483; Older adult sample NR (18–76 years)	Food delivery
11	Roh and Park 2019 [44]	Republic of Korea **** (South Korea)	Total sample n = 500; Older adult sample n = 50 (10%) aged 50–59 years.	Food delivery
12	Sharma et al. 2021 [45]	India **	Total sample n = 440; Older adult sample NR (n = 17, 3.9% aged ≥ 46 years).	Food delivery
13	Tandon et al. 2021 [16]	US ****	Total sample n = 335; Older adult sample n = 17 (4.8%) aged 51–55 years; n = 13 (3.7%) aged 56–60 years.	Food delivery
14	Trivedi et al. 2023 [46]	India **	Total sample n = 121; Older adult sample n = 3 (2.5%) aged ≥ 55 years.	Food delivery
15	Wan et al. 2022 [47]	US ****	Total sample n = 36,846; Older adult sample 100% aged ≥ 50 years ^b^	Not specific ^c^
16	Wang et al. 2022 [19]	China ***	NR ^d^	Food delivery
17	Yap et al. 2022 [48]	Malaysia ***	Total sample n = 302; Older adult sample 100% aged ≥ 60 years ^b^	Food delivery (grocery shopping/delivery)
18	Zanetta et al. 2021 [18]	Brazil ***	Total sample: n = 945; Older adult sample n = 78 (8.3%) aged 50–59 years; n = 43 (4.6%) aged ≥ 60 years.	Food delivery
19	Zhang et al. 2023 [49]	China ***	NR ^d^ (Percentage aged ≥ 60 years: 3.8–50.4%)	Food delivery

This table presents information from the original research studies included in this review. The authors categorized each country as low-, lower middle-, upper middle-, or high-income based on the World Bank’s country classification for development [31]. The sample size is for the analytic sample. The studies did not use the same definition of older adults or report the age distribution in the same way. For studies that did not report the age distribution of study participants, the table shows the age range or other descriptive information about the older adults included in the study. The type of app is as reported in the publication. ^a^ Study was a qualitative study with in-depth interviews. ^b^ 100% of the sample was older adults (50 years and older). ^c^ Studies focused on internet or smartphone use for various activities but did not focus on one type of app. ^d^ Study focused on food insecurity and sampled communities and not people. No data on individual age. ** Lower middle-income country. *** Upper middle-income country. **** High-income country. NR: Not reported; US: United States.

**Table 3:** Topics of the included studies with older adults related to FDAs and food insecurity.

	Abbreviated Citation	Included Older Adults (≥50 Years)	Focused on Older Adults (100% of Sample)	General Technology or App, Including Delivery Services	Included an Online Food Delivery (OFD) or Food Delivery App (FDA)	Related to Food Insecurity	Related to Nutritional Health
1	Apostolidis et al. 2021 [36]	X		X		X	
2	Bakre et al. 2022 [37]	X			X	X	
3	Belarmino et al. 2021 [17]	X			X		
4	Busch et al. 2021 [7]	X	X	X			
5	Dana et al. 2021 [38]	X			X		
6	Kaplan et al. 2023 [39]	X			X		
7	Kaur et al. 2021 [40]	X			X		
8	Köttl et al. 2021 [41]	X		X			
9	Liu et al. 2023 [42]	X			X		
10	Portingale et al. 2023 [43]	X			X		X ^a^
11	Roh & Park 2019 [44]	X			X		
12	Sharma et al. 2021 [45]	X			X		
13	Tandon et al. 2021 [16]	X			X		
14	Trivedi et al. 2023 [46]	X			X		
15	Wan et al. 2022 [47]	X	X	X			
16	Wang et al. 2022 [19]				X	X	
17	Yap et al. 2022 [48]	X	X		X		
18	Zanetta et al. 2021 [18]	X			X		
19	Zhang et al. 2023 [49]				X	X	

Table shows the included original research studies, all journal articles, included in this review. Research reports (n = 2) related to older adults and technology use in general were excluded. ^a^ Study included nutrition-related outcomes of eating behaviors.

## Data Availability

The data are from previously published studies and publicly available datasets.
